# County-level heat vulnerability of urban and rural residents in Tibet, China

**DOI:** 10.1186/s12940-015-0081-0

**Published:** 2016-01-12

**Authors:** Li Bai, Alistair Woodward, Cirendunzhu, Qiyong Liu

**Affiliations:** State Key Laboratory of Infectious Disease Prevention and Control, Collaborative Innovation Center for Diagnosis and Treatment of Infectious Diseases, National Institute for Communicable Disease Control and Prevention, Chinese Center for Disease Control and Prevention, 155 Changbai Road, Changping District Beijing, 102206 P. R. China; School of Population Health, University of Auckland, Private Bag 92019, Auckland, 1142 New Zealand; Tibet Center for Disease Control and Prevention, 21 Linkuo North Road, Lhasa, Tibet 850000 P. R. China; Shandong University Climate Change and Health Center, 44 WenHua Road, Jinan, 250012 Shangdong P. R. China

**Keywords:** Tibet, Climate change, Heat, Vulnerability, Urban, Rural, Adaption

## Abstract

**Background:**

Tibet is especially vulnerable to climate change due to the relatively rapid rise of temperature over past decades. The effects on mortality and morbidity of extreme heat in Tibet have been examined in previous studies; no heat adaptation initiatives have yet been implemented. We estimated heat vulnerability of urban and rural populations in 73 Tibetan counties and identified potential areas for public health intervention and further research.

**Methods:**

According to data availability and vulnerability factors identified previously in Tibet and elsewhere, we selected 10 variables related to advanced age, low income, illiteracy, physical and mental disability, small living spaces and living alone. We separately created and mapped county-level cumulative heat vulnerability indices for urban and rural residents by summing up factor scores produced by a principal components analysis (PCA).

**Results:**

For both study populations, PCA yielded four factors with similar structure. The components for rural and urban residents explained 76.5 % and 77.7 % respectively of the variability in the original vulnerability variables. We found spatial variability of heat vulnerability across counties, with generally higher vulnerability in high-altitude counties. Although we observed similar median values and ranges of the cumulative heat vulnerability index values among urban and rural residents overall, the pattern varied strongly from one county to another.

**Conclusions:**

We have developed a measure of population vulnerability to high temperatures in Tibet. These are preliminary findings, but they may assist targeted adaptation plans in response to future rapid warming in Tibet.

**Electronic supplementary material:**

The online version of this article (doi:10.1186/s12940-015-0081-0) contains supplementary material, which is available to authorized users.

## Background

Tibet of China has been identified as one of areas most vulnerable to climate variability and change in the world [1, 2]. Temperatures on the Tibetan plateau have been increasing by as much as 0.50 °C per decade during the past 30 years [3], a much faster rate of change than has been observed in China, or in Asia generally. In the 2009 summer, maximum temperatures in Lhasa, the capital city of Tibet reached 30.4 °C. Before this, the highest record was 29.9 °C in 1971. Stronger warming trends were observed in winters, with a significant increase of warm winters after 2000 [4]. Warming appears to be positively associated in Tibet with elevation [1]. As a result of these changes, heat has become a new climatic threat in Tibet, although Tibetans still experience periods of extreme cold [4].

The adverse impacts of short-term exposure to ambient high temperatures on human health have been reported in China and worldwide [5–11]. In Tibet, we carried out the first studies on heat-related mortality and morbidity using time-series analyses. For mortality effects, we found those at greater risk of dying on very hot days included the elderly and men, but the effects of temperature on all-cause mortality were not consistent across all study counties [12]. For morbidity effects [13], we found that hot temperatures were more strongly associated with hospital admissions in Lhasa than were cold temperatures. Heat effects were associated with increases of total emergency room visits, hospital admissions for non-accidental diseases, renal diseases and respiratory disease. Again, those ≥ 65 years of age and males were more likely to be affected during high temperature days compared with young people and females . In a cross-sectional survey of 619 urban residents in Lhasa, we found widespread awareness in the local population of rising temperatures and their effects on health, and nearly 40 % reported that they had experienced heat-related symptoms during hot summers [14].

These results, together with climate models that project further warming in the coming decades [3] indicate that action must be taken to minimize the downside health effects of heat, especially among those who are most vulnerable, in the unique high-elevation setting of Tibet. So far, no heat adaptation initiatives have been developed in Tibet. Some of the special features of Tibet that limit its ability to address heat-related health risks include: extremely harsh geographic and environmental conditions, poorly developed public health infrastructure, low coping capacity of the health sector, lack of effective mechanisms for intersectoral collaboration, the distinctive effects of high altitude on health conditions, and the large fraction of the population with low educational attainment. As a first step in responding to these challenges, we aimed to provide information on the vulnerability of populations to heat, in a spatially explicit form.

Elsewhere, studies have mapped heat vulnerability using variables drawn from the literature, but mainly in developed countries. Principal Component Analysis (PCA), a common technique for finding patterns in data of high dimension, has often been used in these studies to construct a composite index. For example, Wolf and McGregor [15] developed a heat vulnerability index in the City of London, UK using 11 indicators from census data. The study found that areas with high sensitivity scores coincided frequently with areas that experienced especially high temperatures. Reid et al. [16] applied a factor analysis with 10 pre-selected vulnerability variables and found cities on the United States west coast and in New England, and generally the downtown areas of large urban centers, to be the places most vulnerable to heat. A similar approach was followed by Harlan et al. [17] for a heat vulnerability assessment in Maricopa County, Arizona, USA. Three factors including socioeconomic vulnerability, elderly/isolation, and unvegetated area explained 79.8 % of the variance in the original dataset in this study.

Most of these studies limited their analysis to urban areas, because those living in urban areas are generally found to be at higher risks of death due in part, perhaps, to the higher temperatures which result from the urban heat island effect [18]. However, some report the relative increase in mortality during heat events was greater in suburban and rural counties, indicating that rural populations may exhibit patterns of vulnerability different from those of urban populations [19]. In China, social, cultural and economic features often vary strongly between city dwellers and those in rural areas. For example, urban areas tend to be relatively modernized with higher socioeconomic level and better infrastructure, while many rural areas still rely heavily on agriculture and have poorly developed health facilities. These factors may also contribute to the different patterns of population vulnerability. Tibet includes commercial and densely populated towns as well as dispersed rural populations in remote villages. Therefore, our study aimed to separately develop and map county-level heat vulnerability indices for urban and rural populations, using variables that have been reported previously to increase vulnerability to heat.

## Methods

### Study population and data sources

**Fig. 1:**
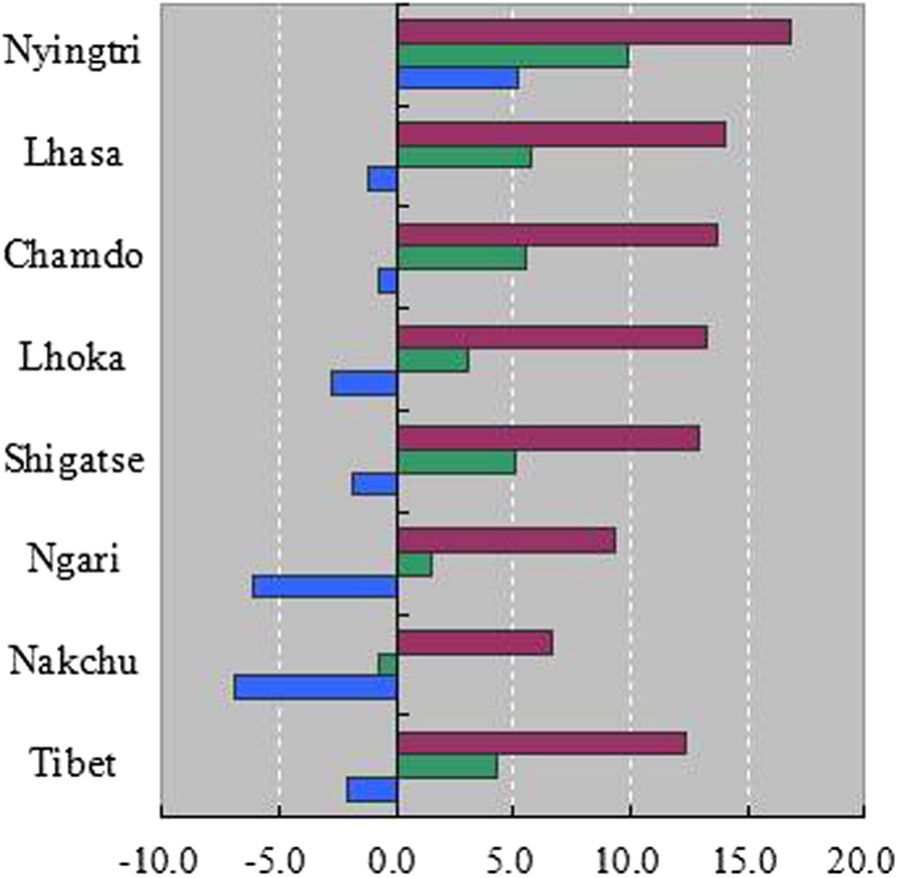
Temperatures in seven regions of Tibet during 1970–2013 (Purple, green and blue represent maximum, mean and minimum temperatures, respectively)

Tibet Autonomous Region, with an average altitude of more than 4,000 meters, is located in the southwest of China, with a land area of 1.22 million square kilometers. There are 74 county-level administrative units in Tibet. In 2010 the population of Tibet numbered 3,002,165 (77.3 % rural residents; 22.7 % urban residents). The region has a complex climate with distinct dry and wet seasons, characterized by cold, dry conditions in the northwest and warm, wet weather in southeast areas. Due to its unique geographic location, Tibet experiences relatively small yearly variations in air temperature, but there are large diurnal swings in temperature. Figure 1 shows average maximum, minimum and mean temperatures in seven regions in Tibet during 1970 to 2013. Nyingtri has the lowest elevation and highest average temperatures, while the lowest temperatures are observed in Nakchu, with a highest average altitude of more than 4,500 meters.

We selected 10 county-level demographic and socioeconomic variables which have been associated with mortality and morbidity during extremely hot days. The variables were chosen according to 1) previous literature, 2) our recent studies in Tibet and 3) data availability. They included measures of advanced age, loss of labor ability, education, living alone, poverty and small dwellings. Table 1 shows the source, definitions and units of these variables. All selected data are available for all counties, except for Shuanghu County. Shuanghu has very few residents due to extreme altitude of more than 5,000 meters, and was recognized as a county only until 2013.

**Table 1:** Sources and definitions of ten selected heat vulnerability variables

Source (Year)	Variable name	Definition ^*a*^
Tibet Census (2010)	Age ≥60	Percent population ≥ 60 years of age
	Loss of labor ability	Percent population losing labor ability because of physical or mental diseases
	Illiterate	Percent urban population ≥ 15 years of age knowing less than 2000 words
		Percent rural population ≥ 15 years of age knowing less than 1500 words
	Living alone	Percent population living alone
	Age ≥60 living alone	Percent population ≥ 60 of age living alone
	Households with only one room	Percent households having only one room which is not a toilet, kitchen or living room
	Households ≤ 8 m^2^ living spaces	Percent households with less than 8 m^2^ gross floor area
Minimum Living Allowances, Ministry of Civil Affairs (2010)	Low income	Percent population receiving the minimum living allowances
	Low income among seniors	Percent elderly population receiving the minimum living allowances
	Low income households	Percent households receiving the minimum living allowances

^***a***^ Definitions of all variables are same for both urban and rural population in Tibet expect for illiterate persons

Higher mortality and morbidity among the elderly, especially those older than 65 years, during extreme heat have been reported elsewhere [20] and observed also in our recent epidemiological studies in Tibet [12, 13]. However, in this assessment, we defined the elderly as those ≥ 60 years of age rather than ≥ 65 years which is the cut-point commonly applied in other developed countries [16, 17], because overall life expectancy in Tibet (68.17 years) is still relatively low [21].

Physical disabilities and pre-existing diseases such as cardiovascular [22], cerebrovascular [23] diabetes [24] and respiratory diseases [13] may increase human susceptibility to heat-related illnesses and death. However, information on the prevalence of these conditions is often lacking. For example, Reid et al. [16] included only diabetes prevalence in their heat vulnerability assessment, as there are no robust comprehensive data on the prevalence of other chronic conditions in the United States. In Tibet also, such variables are not currently available at a county level. We used, instead, the percentage of the population who have partly or totally lost their labour ability due to mental or physical diseases.

We included illiteracy rather than low education as a vulnerability variable. Others have reported that those with low education attainments have a greater vulnerability to weather-related mortality [25, 26]. Nearly all previous assessments considered low education (e.g. less than high school diploma [16, 17]) as an important vulnerability factor. In Tibet, we found that illiterate people tended to be more affected by heat than those who were literate [12]. It should be noted that literacy rates in Tibet have increased dramatically over recent decades. However, the proportion of the population that has never attended school is still high, especially among rural residents.

Social isolation or living alone has been identified as a vulnerability factor in heat events [22, 27]. Isolated seniors who live alone tend to be at even higher risk of dying during heat waves [27]. Unlike other more developed parts of China with a rapidly increasing number of one-senior households, few elderly persons live alone in Tibet (Table 2). However, in line with previous vulnerability studies [16, 17], we included both the percentage of the total population living alone and the fraction of elderly living alone.

**Table 2:** Means, standard deviations (SDs), and Spearman’s correlations for vulnerability variables for urban and rural residents in 73 counties of Tibet

	Age ≥ 60	Loss of labor ability	Illiterate	Living alone	Age ≥ 60 living alone	Low income	Low income among seniors	Low income house-holds	House-holds with only one room	House-holds ≤ 8 m^2^ living spaces
*Urban Residents*										
Mean	5.44	0.23	19.90	7.35	0.47	7.55	1.20	14.20	21.86	5.28
SD	1.69	0.19	10.54	4.72	0.41	7.09	1.59	16.90	12.73	4.46
Minimum	1.25	0.00	2.50	0.90	0.00	0.35	0.01	0.16	0.83	0.17
Maximum	10.14	1.04	63.12	21.30	2.65	31.71	6.82	92.79	61.07	19.89
Age ≥60	1.00									
Loss of labor ability	0.54**	1.00								
Illiterate	0.35**	0.21	1.00							
Living alone	−0.04	0.04	−0.19	1.00						
Age ≥60 living alone	0.40**	0.403**	0.06	0.45**	1.00					
Low income	0.02	−0.12	0.21	−0.15	−0.11	1.00				
Low income among seniors	0.13	0.02	0.19	−0.10	−0.11	0.68**	1.00			
Low income households	0.11	−0.05	0.27*	−0.31**	−0.15	0.89**	0.74**	1.00		
Households with only one room	−0.19	−0.24*	−0.15	0.27*	−0.04	−0.16	−0.35**	−0.35**	1.00	
Households ≤ 8 m^2^ living spaces	−0.08	−0.13	−0.12	−0.04	0.02	−0.07	−0.20	−0.22	0.63**	1.00
*Rural Residents*										
Mean	8.29	0.45	36.38	2.04	0.40	10.17	3.09	13.29	9.93	4.84
SD	1.32	0.16	12.36	1.09	0.24	3.03	2.85	5.91	8.99	5.40
Minimum	5.39	0.05	14.04	0.46	0.05	1.45	0.07	1.50	0.49	0.13
Maximum	11.37	1.08	70.48	5.87	1.42	17.47	11.19	30.05	51.52	28.48
Age ≥60	1.00									
Loss of labor ability	0.04**	1.00								
Illiterate	−0.30**	−0.28*	1.00							
Living alone	0.02	0.22	−0.24*	1.00						
Age ≥60 living alone	0.13	0.19	0.06	0.75**	1.00					
Low income ^*a*^	0.16	−0.16	0.13	−0.15	0.05	1.00				
Low income among seniors	−0.03	−0.10	−0.06	−0.08	−0.02	0.69**	1.00			
Low income households	0.23*	0.03	0.07	−0.16	0.02	0.59**	0.39**	1.00		
Households with only one room	−0.36**	−0.21	0.38**	0.35**	0.34**	−0.33**	-.30**	−0.27*	1.00	
Households ≤ 8 m^2^ living spaces	−0.18	−0.23*	0.33**	0.24*	0.26*	−0.25*	−0.25*	−0.10	0.74**	1.00

*p ≤ 0.05. **p ≤ 0.01

^*a*^ Low income is defined as individuals, seniors and households receiving the minimum living allowances

People receiving low income not only suffer from poorer health in general, but also experience higher mortality during hot days [28, 29]. Poverty levels used in previous vulnerability studies [16, 17, 30] are usually country-specific. The 2010 Tibet Census did not include questions on income. Instead, we collected county-level data on Minimum Living Allowances in 2010 from the Ministry of Civil Affairs, China. We extracted numbers of total population, the elderly and all households that receive the minimum living allowances in urban and rural areas in Tibet.

Small living spaces and poor dwelling conditions are risk factors of heat-related health conditions. One study in Chicago included dwelling-related variables (e.g. median room number and proportion of housing units with only one room) in a social vulnerability index for heat [31]. Similarly, we used in this analysis the percentages of households with only one room and households with less than 8 m^2^ gross floor area.

Consistent with previous similar studies [15–17, 30–32], we did not include sex as a variable, although we have observed previously that Tibetan males are more sensitive than females to extreme heat. The reason is the proportion of males varies little across counties and therefore cannot contribute materially to differences in vulnerability at the population level. Similarly, we did not consider ethnic groups, as 90.48 % of the overall population in Tibet is ethnic Tibetan and only a few counties include small proportions of other ethnic groups.

### Data analysis

The 10 variables selected for analysis are described in Table 2. We carried out Spearman’s correlation analysis to examine the relations between variables. Following a method by Reid et al. [16], we carried out a principal components analysis (PCA) using varimax rotation. PCA is a statistical method to reduce the original dataset to smaller sets of new, independent variables (components). Four principal factors were identified (eigenvalues > 1) for urban and rural residents. Standardized scores (mean = 0; SD = 1) were calculated for each factor and assigned to each county. Each factor score was divided into six categories based on standard deviations. Each category was assigned scores: 1 (≥2 SD below mean), 2 (1–2 SD below mean), 3 (<1 SD below mean), 4 (<1 SD above mean), 5 (1–2 SD above mean), 6 (≥2 SD above mean). We then created a heat vulnerability index that results from summing the integer scores for all four factors for each county. The cumulative vulnerability index values were then mapped using ArcMap 10. 

The analysis was carried out for urban and rural residents separately. The Chinese government announced official definitions of urban and rural area for the purpose of statistics in 2008. The census-defined urban area includes city districts and towns, and rural are defined as all areas rather than city districts and towns. Populations in a county may include urban residents living in a town and rural residents living in villages. All statistical analyses were performed using the R software (version 3.0.1). PCA was performed through “prcomp” package.

## Results

Table 2 shows high correlations between some variables. For both study populations, PCA yielded four factors with similar structure, as shown in Table 3. The components for urban and rural residents explained 77.7 % and 76.5 % respectively of the variability in the original vulnerability variables. For rural residents, two variables related to small dwellings loaded with illiteracy to form factor 1, but they were an independent factor for urban residents. Three aspects of low income were identified as factor 1 for urban residents, but as factor 2 for rural ones. Indicators of social isolation (individuals and seniors living alone) were independently represented as factor 3 in both urban and rural residents’ vulnerability index. The variable ≥ 60 years of age loaded with loss of labor ability to make up factor 4 for the rural population, whereas for those in urban settings, the two variables along with illiteracy were identified as factor 2.

**Table 3:** Principal components analysis of heat vulnerability variables for urban and rural residents in 73 counties of Tibet

	Factor loading							
	Urban				Rural			
	Factor 1: Poverty	Factor 2: Elderly/Fragile health/Illiterate	Factor 3: Social isolation	Factor 4: Small dwelling	Factor 1: Illiterate /Small dwelling	Factor 2: Poverty	Factor 3: Social isolation	Factor 4: Elderly/Fragile health
Age ≥60	0.05	0.87	0.10	−0.09	−0.23	0.12	0.00	0.80
Loss of labor ability	−0.16	0.78	0.10	−0.25	−0.18	−0.05	0.20	0.74
Illiterate	0.47	0.55	−0.25	0.18	0.79	0.12	−0.33	−0.09
Living alone	−0.12	−0.13	0.90	0.01	−0.04	−0.17	0.91	0.03
Age ≥60 living alone	−0.08	0.39	0.80	0.12	0.40	0.15	0.70	0.34
Low income	0.95	0.00	−0.08	0.10	−0.04	0.94	−0.11	0.08
Low income among seniors	0.76	−0.08	0.05	−0.28	−0.31	0.78	0.13	−0.34
Low income households	0.93	0.06	−0.21	−0.07	0.22	0.68	−0.14	0.34
Households with only one room	−0.13	−0.28	0.20	0.78	0.76	−0.22	0.34	−0.30
Households ≤ 8 m^2^ living spaces	−0.03	0.00	−0.05	0.88	0.76	−0.05	0.28	−0.22

Absolute values > 0.5 are the most significant loadings on that factor

We observed the same median values and similar ranges of the cumulative heat vulnerability index values between the two study populations (Additional file 1). Numbers of counties above the mean value for all four factors are 28 and 24 respectively for urban and rural residents (Additional file 1). Ten counties have the same vulnerability index scores for those living in urban and rural areas. In absolute terms the vulnerability of the two populations varies by county: Gyantse County of Shigatse Area had the highest cumulative vulnerability index value for urban residents, and this was greater than the value for the rural populations in the same county. Tsochen County of Ngari Area had the highest vulnerability scores for rural residents, while the vulnerability of the urban population in this country was relatively low.

We also calculated each area’s mean cumulative heat vulnerability index value (Additional file 1). Lhasa had the second-highest vulnerability of urban people, but the lowest value for rural residents. This is mainly driven by the fact that Lhasa has the highest score of factor 2 among urban population, but very low value of the same factor among rural population. In contrast, Ngari had the highest score for rural residents, but the lowest vulnerability among those in urban areas. In Nyingtri Area, the difference between heat vulnerability of the two populations is the smallest.

**Fig. 2:**
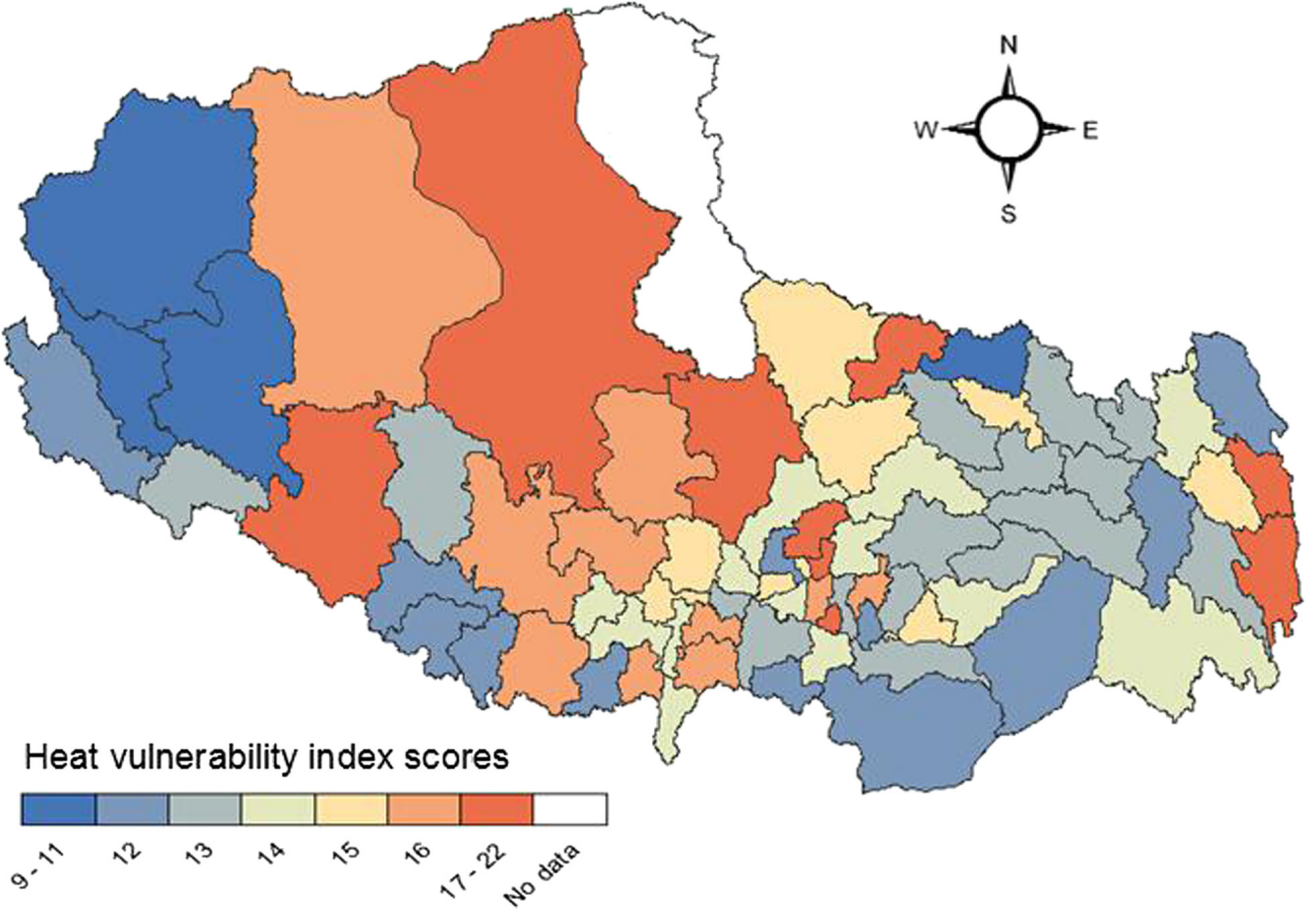
Map of cumulative heat vulnerability by county for urban residents in Tibet

**Fig. 3:**
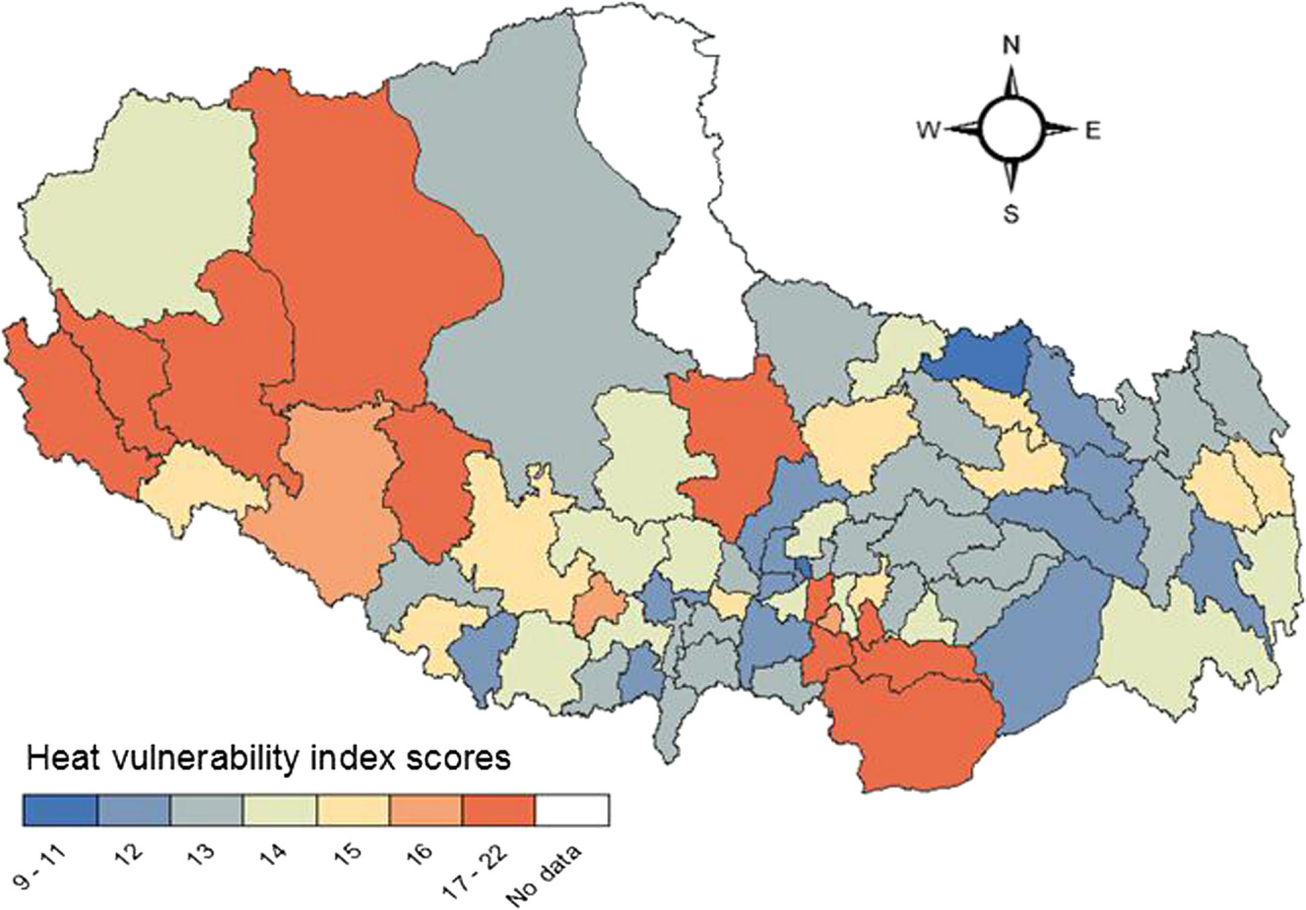
Map of cumulative heat vulnerability by county for rural residents in Tibet

Figure 2 and 3 show the geographic distribution of the cumulative vulnerability index for urban and rural residents respectively. In urban areas, higher heat vulnerability was seen in the middle and Northwest and east of Tibet. For rural areas, clustering of high vulnerability was found in Western, central and Southeastern Tibet. Clustering of lower vulnerability was seen in Nyingtri Area in Southeastern Tibet for both the two populations.

## Discussion

This is the first study assessing and mapping heat vulnerability of urban and rural populations, in the Tibet Autonomous Region of China. Our analysis was based on 10 factors which may increase human vulnerability to heat according to previous epidemiologic research, and we applied similar statistical methods to those used by Reid et al. [16]. Overall, our study shows that the relative vulnerabilities of the two study populations varies across counties, with generally higher (and more adverse) scores for vulnerability of rural populations in the central Tibet, and for urban residents in the West and Southeast. Particular attention should be paid to those high-elevation areas in South Tibet. They not only display relatively higher population vulnerability (due to higher proportions of relevant demographic risk factors), but also are warming more quickly than parts of Tibet at low altitude.

There is a growing body of work on spatial heat vulnerability indices. However, most of the studies carried out in the past were conducted in cities of developed countries such as the UK [15], the United States [16, 31], Australia [32] and Canada [30]. Little is known about spatial variability of heat vulnerability in areas of developing countries like China and there have been no relevant reports previously from a high-elevation setting. The Tibetan plateau may not experience such extreme heat events as other inland provinces of China, but the temperature in Tibet is increasing more quickly than other areas of China [1, 4]. Moreover, we have already noted that some sub-populations including the elderly, men and illiterate persons are at higher risks of dying or being sick during high temperature days [12, 13]. The vulnerability approach could be a useful tool for a rapid developing Tibet to quickly identify potential areas for heat-health action plans. This kind of literature-based assessment does not require us to wait until all determinants of human vulnerability are described comprehensively in regions which lack reliable long-term health data.

The factor structure of derived heat vulnerabilities indices is generally the same for the two study populations in Tibet. One exception which should be noted is the configuration of factor loadings for the illiteracy variable. It loaded with small living spaces to form an independent factor in rural populations, but with variables of advanced age and those who lost labor abilities in the index of urban residents. For those living in rural villages in Tibet, higher proportions of elderly residents and those living in small dwellings tended to occur in the same counties. This may be explained by a tendency for the oldest sections of the population to live in old and crowed dwellings in poor and remote neighborhoods in China. In contrast, most of the urban residents in small living spaces are likely to be younger people who have been drawn to the cities or towns and are sharing rental dwellings. In the urban residents’ index, variables for illiteracy, the elderly and loss of labor ability loaded on the same factor, suggesting that illiterate residents living in cities and towns tend to be older and experience mental or physical disabilities.

In this study we created cumulative heat vulnerability indices for urban and rural populations separately. We suggest that this approach may enable better targeted assignments of adaptive interventions and resources, because conditions for urban and rural residents in the same county in Tibet may be quite different. For example, in Ngari Area, we observed the highest vulnerability score of rural populations, but the lowest for urban residents (Additional file 1). Ngari Area, with an average altitude of 4500 m, has one of the lowest population densities in the world due to its extreme altitude and very harsh natural environment. Rural residents in this area tend to live in remote villages, and are further disadvantaged by limited access to education, low income, poor housing quality and living conditions. The area with the lowest vulnerability values of urban residents is also found to be Ngari Area, possibly explained by the fact that it has the lowest illiteracy rate among urban people in all Tibet. Educational facilities and conditions have been improved dramatically over recent decades in Ngari by the Chinese government’s substantial investment. However, most of the new schools are located in towns, and greater investments in education in rural and remote areas in Tibet are needed to minimize educational inequality and vulnerability to poor health.

Chinese governments have designed and implemented a variety of research projects, policies and plans to better build capacity to cope with meteorological disasters and long-term climate change in Tibet, while the health implication of climate change are relatively neglected. An Assessment Report of Climate Change Impacts in Tibet Autonomous Region has been published by the National and Tibet Climate Centers to better understand current and future adverse effects of climate variability and change. This report recognizes the adverse impacts of a rapidly warming climate in Tibet including snow line rising, glacial recession, changes in river levels, frozen soil layer movements towards the north, grassland degradation, increasing plant diseases and pests, decreasing biological diversity and more meteorological disasters. However, there are no human health data nor is an assessment of health risks included in the report. Another example is the development and implementation of the “Programme to address climate change in Tibet Autonomous Region” by the Development and Reform Commission and Meteorological Administration of Tibet. The Plan did not consider the public health challenges of a changing climate, although it identified systematic strategies to deal with climate change effects on agriculture, livestock, forest, water resources, industry and natural ecosystems. We suggest that better knowledge of the range and magnitude of temperature-sensitive health outcomes is the first important step to increase awareness at the local government level of health implications of climate change.

The limited capacity of public health departments in Tibet creates further challenges. The local Center of Disease Control and Prevention (CDC) is the major institution working in the field of diseases control and public health managements, and is supposed to be the lead agency when it comes to developing public health components of heat preparedness measures in Tibet. There are a number of barriers that make it difficult for Tibet CDC to manage the health risks related to rising temperatures. Chronic staff shortage, insufficient expertise and limited technical skills of current staff are the major constraints. According to Tibetan Statistics in 2008, despite dramatic increases in income levels, savings, educational attainment and dwelling conditions over past two decades, the number of public health technical personnel per 1000 persons has fallen from 3.39 in 1990 to 3.02 in 2008. Options for building capacity in the health sector include increasing the number of public health personnel, and improving education and training of the current work force. The capacity to develop adaptation policies and measures in health sections is restricted also by limited information on health impacts of climate variability due to an absence of reliable health data in Tibet. At present, there are only five counties that have been selected to carry out long-term surveillance of death and prevalence of chronic diseases in Tibet. Long-term hospital-based data are also limited due to very uneven spatial distributions of hospitals and lack of well-established electronic medical record systems. More surveillance locations are required to collect more valid and comprehensive health data for future research and intervention planning. Also, sharing access to existing relevant data sets should be improved to meet needs of sustainable intersectoral collaboration and actions.

The heat vulnerability indices we developed have a number of limitations that users need to keep in mind when using the results for decision-making. Our analysis was limited by the lack of data for other important determinants of heat-health vulnerability. We did not include exposure variables which are frequently used in other assessments of this kind, such as land cover and surface temperature. Extreme urban heat is still not very common in Tibet except in a few locations such as Chengguan District of Lhasa and uneven spatial resolution of surface temperature datasets permits only intra-urban heat vulnerability assessments [33]. Furthermore, surface temperature and green spaces are not the only factors which determine air temperature and indoor temperatures. Unlike other studies, we did not include a measure of home air-conditioning (AC) although it is known access to air-conditioning may protect against heat-related illnesses and deaths [22, 34, 35]. The reasons are that, first, these data are not available at county-level and, second, air-conditioning at home is uncommon in Tibet (in 2008 it was estimated that only 3 % of households had AC) [36]. Similarly, we did not include a measure of use of cooling fans, as this information is not available. Besides, although many studies have indicated that individuals in occupations that entail exposure to high temperatures outdoors are more likely to develop heat-induced diseases, we were unable to include a measure of occupation in this study. Housing factors (e.g. dwelling age, the number of floors, construction types) may also increase the likelihood of exposure to severe heat and influence heat-health risks. However, these data were not included in our analysis, as there are too many missing values for most of the remote counties. Another important limitation related to data unavailability in this study is lack of sensitivity analysis using alternative variables.

Given the lack of information on other domains of vulnerability, our indices were mainly based on demographic variables. We observed different demographic patterns associated with heat vulnerability between urban and rural populations. For instance, illiteracy is strongly associated with household size in rural settings but matches closely with age and disability in urban settings. These findings indicate that the population variables which can best represent heat vulnerability are different in urban and rural areas. Nevertheless, the 10 variables in this study were based on our epidemiological studies in Tibet as well as validated vulnerability indices in previous literature. Despite their imperfections we suggest the results in this study provide important and timely information to policy makers and can be used along with local meteorological records in developing heat adaptation plans.

Following Reid et al. [16], we created heat vulnerability indices without weighting factors. The use of a composite index without weights is not ideal, and future studies may explore differential weighting of the variables based on closer understanding of heat-health vulnerability pattern in Tibet. The composite indices in this analysis have not been validated with local health data since this information is not currently available. Validation is certainly an important component in vulnerability assessment studies [37], but the next step, in the absence of comprehensive local data sets, could be to consult local Tibetan stakeholders and relevant professionals, obtain feedback on the heat vulnerability maps, and modify as appropriate variable selection and other aspects of the analysis.

## Conclusions

In this analysis, we separately developed and mapped county-level heat vulnerability indices for urban and rural populations in Tibet. Heat vulnerability varies spatially on county scales. For both rural and urban populations, we observed higher heat vulnerability in higher-altitude counties compared with counties with relative low altitude, due to higher proportions of vulnerable groups in remote high-elevation areas. However, we found the relative vulnerability of rural and urban residents may differ between counties. At present, no adaptive initiatives have been taken to reduce the heat-related health risks in Tibet. Our findings provide the preliminary information to public health sections to prepare more targeted adaptation strategies for urban and rural residents in Tibet, while future studies are required to validate the indices with local health outcome data.

## Additional file

Additional file 1:
**Proportions and vulnerability scores of urban and rural residents in each county.** (DOC 157 kb)
