# Supplementary material for: County-level heat vulnerability of urban and rural residents in Tibet, China
Source: Environ Health. 2016 Jan 12;15:3. doi: 10.1186/s12940-015-0081-0 (PMC4711018; doi:10.1186/s12940-015-0081-0)
Supplement: Additional file 1: — Proportions and vulnerability scores of urban and rural residents in each county. (DOC 157 kb) [file 12940_2015_81_MOESM1_ESM.doc]

**Additional file 1. Proportions and vulnerability scores of urban and rural** residents in each county

|  | **Population (%)** | | **Factor 1** | | **Factor 2** | | **Factor 3** | | **Factor 4** | | **Vulnerability scores** | | **Difference*** |
| --- | --- | --- | --- | --- | --- | --- | --- | --- | --- | --- | --- | --- | --- |
|  | **Urban** | **Rural** | **Urban** | **Rural** | **Urban** | **Rural** | **Urban** | **Rural** | **Urban** | **Rural** | **Urban** | **Rural** |
| Mean |  |  |  |  |  |  |  |  |  |  | 14.15 | 14.04 |  |
| Median |  |  |  |  |  |  |  |  |  |  | 14.00 | 14.00 |  |
| SD |  |  |  |  |  |  |  |  |  |  | 2.07 | 2.02 |  |
| Range |  |  |  |  |  |  |  |  |  |  | 10 - 21 | 9 - 21 |  |
| ***Lhasa*** | 43.1 | 56.9 | 3.3 | 3.0 | 4.1 | 2.4 | 3.8 | 3.8 | 3.8 | 3.4 | **14.9** | 12.5 | 2.4 |
| Chengguan | 71.4 | 28.6 | 3 | 3 | 3 | 1 | 4 | 6 | 5 | 1 | **15** | 11 | 4 |
| Lhundup | 5.8 | 94.2 | 6 | 3 | 4 | 4 | 4 | 3 | 3 | 4 | **17** | 14 | 3 |
| Damshung | 11.9 | 88.1 | 3 | 4 | 4 | 2 | 3 | 3 | 4 | 3 | 14 | 12 | 2 |
| Nyemo | 11.3 | 88.7 | 3 | 3 | 4 | 3 | 3 | 3 | 4 | 4 | 14 | 13 | 1 |
| Chushur | 15.5 | 84.5 | 3 | 3 | 5 | 2 | 3 | 3 | 4 | 4 | **15** | 12 | 3 |
| Tolung Dechen | 33.5 | 66.5 | 2 | 2 | 4 | 2 | 3 | 5 | 3 | 3 | 12 | 12 | 0 |
| Taktse | 13.4 | 86.6 | 3 | 3 | 5 | 2 | 6 | 4 | 4 | 4 | 18 | 13 | 5 |
| Medro Gongkar | 9.3 | 90.7 | 3 | 3 | 4 | 3 | 4 | 3 | 3 | 4 | 14 | 13 | 1 |
| ***Chamdo*** | 13.0 | 87.0 | 3.3 | 3.8 | 3.6 | 3.5 | 3.5 | 2.2 | 3.7 | 3.8 | **14.1** | 13.4 | 0.7 |
| Chamdo | 37.8 | 62.2 | 3 | 3 | 4 | 3 | 3 | 3 | 4 | 4 | 14 | 13 | 1 |
| Gyamda | 3.2 | 96.8 | 4 | 4 | 3 | 3 | 2 | 2 | 3 | 4 | 12 | 13 | -1 |
| Gongjo | 7.2 | 92.8 | 4 | 4 | 3 | 4 | 6 | 2 | 4 | 5 | **17** | **15** | 2 |
| Rioche | 12.5 | 87.5 | 3 | 4 | 3 | 3 | 3 | 3 | 4 | 3 | 13 | 13 | 0 |
| Tengchen | 5.9 | 94.1 | 3 | 4 | 3 | 4 | 3 | 2 | 4 | 2 | 13 | 12 | 1 |
| Dayak | 7.6 | 92.4 | 3 | 5 | 5 | 3 | 3 | 2 | 4 | 5 | **15** | **15** | 0 |
| Paksho | 11.7 | 88.3 | 3 | 3 | 3 | 4 | 3 | 2 | 3 | 4 | 12 | 13 | -1 |
| Zogong | 8.1 | 91.9 | 3 | 4 | 4 | 3 | 3 | 2 | 3 | 3 | 13 | 12 | 1 |

**Appendix Table. Proportions and vulnerability scores of urban and rural residents in each county (continued)**

|  | **Population (%)** | | **Factor 1** | | **Factor 2** | | **Factor 3** | | **Factor 4** | | **Vulnerability scores** | |  |
| --- | --- | --- | --- | --- | --- | --- | --- | --- | --- | --- | --- | --- | --- |
|  | **Urban** | **Rural** | **Urban** | **Rural** | **Urban** | **Rural** | **Urban** | **Rural** | **Urban** | **Rural** | **Urban** | **Rural** | **Difference*** |
| Markham | 5.3 | 94.7 | 4 | 4 | 4 | 3 | 6 | 2 | 6 | 5 | **20** | 14 | 6 |
| Lhorong | 12.2 | 87.8 | 3 | 3 | 4 | 4 | 3 | 2 | 3 | 3 | 13 | 12 | 1 |
| Palbar | 8.3 | 91.7 | 3 | 4 | 4 | 5 | 3 | 2 | 3 | 4 | 13 | **15** | -2 |
| ***Lhoka*** | 21.9 | 78.1 | 3.1 | 2.8 | 4.0 | 3.9 | 3.9 | 4.2 | 2.8 | 4.3 | 13.8 | **15.3** | -1.5 |
| Nedong | 51.4 | 48.6 | 3 | 2 | 3 | 3 | 4 | 5 | 3 | 4 | 13 | 14 | -1 |
| Danang | 5.1 | 94.9 | 4 | 2 | 6 | 4 | 4 | 5 | 2 | 6 | **16** | **17** | -1 |
| Gonggar | 24.5 | 75.5 | 3 | 3 | 5 | 4 | 3 | 3 | 3 | 4 | 14 | 14 | 0 |
| Sangri | 11.2 | 88.8 | 3 | 4 | 4 | 3 | 5 | 3 | 4 | 5 | **16** | **15** | 1 |
| Chong-Gye | 14.4 | 85.6 | 3 | 3 | 5 | 4 | 5 | 4 | 4 | 5 | **17** | **16** | 1 |
| Chosum | 18.8 | 81.2 | 3 | 3 | 3 | 4 | 4 | 5 | 2 | 5 | 12 | **17** | -5 |
| Tsome | 31.3 | 68.7 | 3 | 3 | 5 | 5 | 3 | 4 | 3 | 5 | 14 | **17** | -3 |
| Lhodak | 18.1 | 81.9 | 3 | 3 | 4 | 4 | 3 | 2 | 2 | 4 | 12 | 13 | -1 |
| Lhuntse | 14.2 | 85.8 | 3 | 3 | 3 | 3 | 3 | 4 | 4 | 3 | 13 | 13 | 0 |
| Tsona | 14.9 | 85.1 | 3 | 3 | 4 | 5 | 4 | 5 | 2 | 4 | 13 | **17** | -4 |
| Nakartse | 18.9 | 81.1 | 3 | 3 | 3 | 5 | 4 | 6 | 2 | 4 | 12 | **18** | -6 |
| Gyatsa | 6.7 | 93.3 | 3 | 2 | 3 | 3 | 5 | 4 | 2 | 3 | 13 | 12 | 1 |
| ***Shigatse*** | 17.6 | 82.4 | 4.4 | 3.1 | 3.8 | 4.4 | 3.3 | 3.5 | 3.2 | 2.7 | **14.7** | **13.7** | 1 |
| Shigatse City | 53.1 | 46.9 | 3 | 3 | 4 | 3 | 4 | 3 | 4 | 3 | **15** | 12 | 3 |
| Namling | 6.1 | 93.9 | 5 | 3 | 4 | 5 | 3 | 3 | 3 | 3 | **15** | 14 | 1 |
| Tingri | 11.0 | 89.0 | 4 | 3 | 5 | 3 | 4 | 3 | 3 | 4 | **16** | 13 | 3 |
| Sakya | 6.5 | 93.5 | 5 | 3 | 3 | 5 | 5 | 4 | 3 | 2 | **16** | 14 | 2 |

**Appendix Table. Proportions and vulnerability scores of urban and rural residents in each county (continued)**

|  | **Population (%)** | | **Factor 1** | | **Factor 2** | | **Factor 3** | | **Factor 4** | | **Vulnerability scores** | |  |
| --- | --- | --- | --- | --- | --- | --- | --- | --- | --- | --- | --- | --- | --- |
|  | **Urban** | **Rural** | **Urban** | **Rural** | **Urban** | **Rural** | **Urban** | **Rural** | **Urban** | **Rural** | **Urban** | **Rural** | **Difference*** |
| Lhatse | 12.1 | 87.9 | 3 | 3 | 6 | 4 | 3 | 4 | 2 | 3 | 14 | 14 | 0 |
| Thongmon | 10.9 | 89.1 | 4 | 4 | 4 | 5 | 2 | 4 | 4 | 3 | 14 | **16** | -2 |
| Ngamring | 2.0 | 98.0 | 6 | 4 | 4 | 4 | 3 | 4 | 3 | 3 | **16** | **15** | 1 |
| Tingkye | 9.8 | 90.2 | 4 | 4 | 4 | 5 | 4 | 3 | 4 | 2 | **16** | 14 | 2 |
| Dongpa | 11.6 | 88.4 | 3 | 3 | 4 | 3 | 3 | 3 | 4 | 4 | 14 | 13 | 1 |
| Kyirong | 5.1 | 94.9 | 5 | 2 | 3 | 5 | 3 | 3 | 2 | 5 | 13 | **15** | -2 |
| Nyalam | 7.7 | 92.3 | 4 | 3 | 3 | 4 | 6 | 3 | 3 | 3 | **16** | 13 | 3 |
| Saga | 18.0 | 82.0 | 5 | 3 | 3 | 5 | 2 | 3 | 2 | 2 | 12 | 13 | -1 |
| Gyantse | 3.2 | 96.8 | 6 | 4 | 6 | 5 | 3 | 5 | 6 | 2 | **21** | **16** | 5 |
| Panam | 45.6 | 54.4 | 4 | 2 | 4 | 5 | 3 | 4 | 3 | 2 | 14 | 13 | 1 |
| Rinpung | 24.7 | 75.3 | 3 | 3 | 3 | 6 | 3 | 4 | 3 | 2 | 12 | **15** | -3 |
| Khangmar | 23.5 | 76.5 | 4 | 3 | 2 | 4 | 3 | 3 | 3 | 2 | 12 | 12 | 0 |
| Gampa | 14.6 | 85.4 | 5 | 2 | 2 | 5 | 3 | 3 | 2 | 3 | 12 | 13 | -1 |
| Yatung | 4.9 | 95.1 | 6 | 3 | 4 | 4 | 2 | 4 | 4 | 1 | **16** | 12 | 4 |
| ***Nakchu*** | 16.3 | 83.7 | 3.8 | 4.3 | 3.4 | 3.0 | 3.5 | 3.2 | 4.3 | 3.1 | **15** | 13.6 | 1.4 |
| Nakchu | 39.5 | 60.5 | 3 | 4 | 4 | 4 | 4 | 3 | 4 | 4 | **15** | **15** | 0 |
| Chali | 8.9 | 91.1 | 3 | 4 | 3 | 3 | 4 | 3 | 4 | 3 | 14 | 13 | 1 |
| Dirl | 7.1 | 92.9 | 4 | 4 | 3 | 3 | 3 | 3 | 3 | 3 | 13 | 13 | 0 |
| Nyerong | 7.0 | 93.0 | 6 | 5 | 2 | 3 | 4 | 3 | 5 | 3 | **17** | 14 | 3 |
| Amdo | 16.7 | 83.3 | 3 | 5 | 4 | 3 | 3 | 3 | 5 | 2 | **15** | 13 | 2 |
| Shantsa | 14.3 | 85.7 | 4 | 4 | 3 | 3 | 4 | 4 | 5 | 3 | **16** | 14 | 2 |
| Sokshan | 10.8 | 89.2 | 3 | 4 | 4 | 3 | 3 | 4 | 5 | 4 | **15** | **15** | 0 |

**Appendix Table. Proportions and vulnerability scores of urban and rural residents in each county (continued)**

|  | **Population (%)** | | **Factor 1** | | **Factor 2** | | **Factor 3** | | **Factor 4** | | **Vulnerability scores** | |  |
| --- | --- | --- | --- | --- | --- | --- | --- | --- | --- | --- | --- | --- | --- |
|  | **Urban** | **Rural** | **Urban** | **Rural** | **Urban** | **Rural** | **Urban** | **Rural** | **Urban** | **Rural** | **Urban** | **Rural** | **Difference*** |
| Palgon | 13.9 | 86.1 | 4 | 6 | 4 | 4 | 4 | 3 | 5 | 4 | **17** | **17** | 0 |
| Bachen | 5.0 | 95.0 | 3 | 3 | 2 | 2 | 4 | 2 | 2 | 2 | 11 | 9 | 2 |
| Nima | 3.5 | 96.5 | 5 | 4 | 5 | 2 | 2 | 4 | 5 | 3 | 17 | 13 | 4 |
| ***Ngari*** | 23.0 | 77.0 | 3.0 | 5.0 | 2.6 | 3.4 | 3.3 | 4.6 | 3.4 | 4.3 | **12.3** | **17.9** | -5.6 |
| Purang | 25.5 | 74.5 | 3 | 3 | 2 | 3 | 4 | 5 | 4 | 4 | 13 | **15** | -2 |
| Tsada | 21.7 | 78.3 | 3 | 4 | 3 | 4 | 3 | 4 | 3 | 5 | 12 | **17** | -5 |
| Gar | 60.8 | 39.2 | 2 | 5 | 2 | 3 | 3 | 5 | 4 | 5 | 11 | **18** | -7 |
| Rutok | 20.6 | 79.4 | 3 | 5 | 2 | 3 | 3 | 3 | 2 | 3 | 10 | 14 | -4 |
| Gakyi | 3.3 | 96.7 | 4 | 6 | 1 | 3 | 3 | 5 | 3 | 3 | 11 | **17** | -6 |
| Gertse | 12.5 | 87.5 | 3 | 6 | 4 | 4 | 4 | 5 | 5 | 4 | **16** | **19** | -3 |
| Tsochen | 16.3 | 83.7 | 3 | 6 | 4 | 4 | 3 | 5 | 3 | 6 | 13 | **21** | -8 |
| ***Nyingtri*** | 31.5 | 68.5 | 3.0 | 3.1 | 3.0 | 2.3 | 3.4 | 4.0 | 4.0 | 3.6 | 13.4 | 13.0 | 0.4 |
| Nyingtri | 64.3 | 35.7 | 3 | 3 | 2 | 2 | 3 | 4 | 5 | 4 | 13 | 13 | 0 |
| Kongpo Gyamda | 13.5 | 86.5 | 3 | 3 | 2 | 2 | 4 | 4 | 4 | 4 | 13 | 13 | 0 |
| Miling | 23.8 | 76.2 | 3 | 3 | 3 | 2 | 4 | 4 | 4 | 4 | 14 | 13 | 1 |
| Metok | 18.1 | 81.9 | 3 | 3 | 2 | 2 | 3 | 4 | 4 | 3 | 12 | 12 | 0 |
| Pome | 26.8 | 73.2 | 3 | 3 | 3 | 2 | 3 | 4 | 4 | 3 | 13 | 12 | 1 |
| Zayul | 15.4 | 84.6 | 3 | 4 | 4 | 4 | 3 | 2 | 4 | 4 | 14 | 14 | 0 |
| Namshan | 10.5 | 89.5 | 3 | 3 | 5 | 2 | 4 | 6 | 3 | 3 | **15** | 14 | 1 |

***Differences equal to vulnerability values of urban populations minus those of rural populations**

**The bold means scores higher than the median score of vulnerability**
